# Attribution of smoking to healthcare costs in the postoperative interval

**DOI:** 10.1093/bjsopen/zrae090

**Published:** 2024-08-16

**Authors:** Helene L Gräsbeck, Aleksi R P Reito, Heikki J Ekroos, Juhani A Aakko, Olivia Hölsä, Tuula M Vasankari

**Affiliations:** Pulmonary Unit, HUS Porvoo Hospital, Porvoo, Finland; Doctoral Programme of Clinical Research, University of Helsinki, Helsinki, Finland; Centre for Musculoskeletal Diseases, Tampere University Hospital, Tampere, Finland; Faculty of Medicine and Health Technology, Tampere University, Tampere, Finland; Pulmonary Unit, HUS Porvoo Hospital, Porvoo, Finland; Medaffcon Oy, Espoo, Finland; Medaffcon Oy, Espoo, Finland; Department of Pulmonary Diseases and Clinical Allergology, University of Turku, Turku, Finland; Finnish Lung Health Association (Filha), Helsinki, Finland

## Introduction

A study that included 27 countries revealed that 16.8% of patients undergoing elective surgery developed at least one postoperative complication leading to intensive care^1^. A meta-analysis of 107 studies revealed that smoking is associated with postoperative infections, wound, pulmonary, and neurological complications, and intensive care admission^2^. In a statewide American study, one-quarter of surgical patients were smokers^3^.

Jiménez-Ruiz *et al*.^4^ estimated the cost of preoperative smoking cessation programmes, including medical counselling and drug therapy, such as varenicline (Champix^®^, Pfizer, New York City, NY, USA), bupropion (Zyntabac^®^; Glaxo Wellcome, Burgos, Spain), or transdermal nicotine replacement therapy (Nicotinell, GlaxoSmithKline, London, UK), and revealed that it was vastly outweighed by the savings in healthcare costs^4^.

There are very few studies on the association between current and former smoking and healthcare costs in the postoperative interval.

The aim of this study was to determine whether current and former smoking are associated with increased 90-day postoperative costs due to prolonged length of stay or emergency department visits leading to either patient readmission or discharge. The role of smoking in relation to other risk factors was also explored.

## Methods

### Study population

This was a retrospective cohort study including patients who had undergone surgery between January 2015 and December 2019 in the Finnish hospital district of Helsinki and Uusimaa (HUS). Data were retrieved from HUS Datalake, a database covering local electronic health record systems.

### Outcome

The impact of preoperative smoking status (never smoker, former smoker, and current smoker) on costs was assessed using three gamma regression models with a log-link function. In these models, the outcome was 90-day postoperative healthcare costs due to in-hospital care and emergency department visits leading to either patient readmission or discharge. Age, sex, preoperative smoking status, ASA grade, and chronic diseases as classified in the Charlson co-morbidity index^5^ were explanatory patient variables. As ASA grade I refers to a healthy patient and current smokers are assigned to ASA grade II, ASA grades I and II were combined to prevent the variable from acting as a mediator on the potential causal pathway between smoking and increased costs. The Charlson co-morbidity index was calculated using ICD-10 codes as implemented in the co-morbidity R package^5,6^. Surgical variables were urgency class (elective, urgent, or emergency surgery), time spent in the operating room, and anaesthesia type. The relative importance of the variables in the models was assessed by calculating the partial chi-squared statistic (Wald X)^7^ for each variable and a global importance score based on Shapley values^8^. Higher values indicate greater variable importance in the model and variables that fail to add value to the model have parameters equalling zero (see the *Supplementary Methods* for exclusion criteria, cost calculations, smoking status definition, and statistical analysis).

## Results

The sample included 185 100 surgeries (*Fig. S1*). Of these, 97 724 (52.8%) were performed on never smokers, 36 593 (19.8%) on former smokers, and 50 783 (27.4%) on current smokers (*Table 1*).

**Table 1:** Baseline characteristics

Characteristic	Never smoker (*n* = 97 724)	Former smoker (*n* = 36 593)	Current smoker (*n* = 50 783)
Male	33 085 (33.8)	19 882 (54.3)	23 923 (47.1)
**Age (years), mean(s.d.), median (i.q.r.)**	57(18), 58 (42–71)	63(15), 66 (53–74)	51(16), 53 (38–64)
ASA grade*			
I	22 029 (22.5)	3420 (9.3)	8073 (15.9)
II	43 433 (44.4)	13 428 (36.7)	24 055 (47.3)
III	26 941 (27.6)	15 206 (41.6)	14 987 (29.5)
IV	5122 (5.2)	4403 (12.0)	3470 (6.8)
V	199 (0.2)	136 (0.4)	198 (0.4)
Charlson co-morbidity index*			
0†	63 290 (64.8)	16 004 (43.7)	32 530 (64.1)
1†	26 017 (26.6)	12 655 (34.6)	12 937 (25.5)
2†	6429 (6.6)	5293 (14.5)	3818 (7.5)
3†	1528 (1.6)	1779 (4.9)	1087 (2.1)
4†	384 (0.4)	628 (1.7)	322 (0.6)
≥5†	76 (<0.1)	234 (0.6)	89 (0.2)
Urgency class*			
Elective	54 745 (56.0)	20 081 (54.9)	25 633 (50.5)
Urgent	27 814 (28.5)	11 337 (31.0)	15 960 (30.9)
Emergency (<24 h)	15 165 (15.5)	5175 (14.1)	9190 (18.1)
Anaesthesia type*			
No anaesthesia	268 (0.3)	84 (0.2)	105 (0.2)
Local anaesthesia	5295 (5.4)	2435 (6.7)	2392 (4.7)
Regional anaesthesia or sedation	35 499 (36.3)	12 905 (35.3)	16 658 (32.8)
General anaesthesia	56 662 (58.0)	21 169 (57.8)	31 628 (62.3)
**Time spent in the operating room (h), mean(s.d.), median (i.q.r.)**	2.29(1.49), 1.96 (1.35–2.80)	2.46(1.66), 2.05 (1.38–3.02)	2.32(1.56), 1.95 (1.30–2.87)

Values are *n* (%) unless otherwise indicated. *Percentages have been rounded and might not total 100. †Total number of Charlson co-morbidity index co-morbidities. i.q.r., interquartile range.

In the adjusted gamma regression models 2 and 3, current smokers had significantly increased costs (OR 1.03 (95% c.i. 1.02 to 1.04), *P* < 0.001, for model 2 and OR 1.02 (95% c.i. 1.01 to 1.03), *P* = 0.002, for model 3), but former smokers did not (*Table S1*).

In the prediction of total 90-day postoperative costs by smoking status, former smokers had costs of €3107 (95% c.i. €3068 to €3149) and current smokers had costs of €3287 (95% c.i. €3247 to €3325) in adjusted model 2. The differences compared with never smokers were €−78 (95% c.i. €−116 to €−38, *P* < 0.001) and €102 (95% c.i. €63 to €139, *P* < 0.001) respectively (*Table 2*). In the prediction of total costs by urgency class and smoking status, the lowest costs were found in the reference group never smokers undergoing elective surgery (€2458 (95% c.i. €2435 to €2481)) and the largest costs were found for current smokers undergoing emergency surgery (€5489 (95% c.i. €5409 to €5572), *P* < 0.001) (*Table S2*).

**Table 2:** Predicted total costs by smoking status

		Smoking status		
		Never smoker	Former smoker	Current smoker
Model 1*	Costs (€) (95% c.i.)	4691 (4657,4726)	5823 (5757,5890)	5154 (5096,5210)
	Total events† (95% c.i.)	4.33 (4.31,4.36)	5.30 (5.25,5.35)	4.74 (4.71,4.78)
Model 2‡	Costs (€) (95% c.i.)	3185 (3160,3211)	3107 (3068,3149)	3287 (3247,3325)
	Difference (€) (95% c.i.; *P*)	–	−78 (−116,−38; <0.001)	102 (63,139; <0.001)
	Total events† (95% c.i.)	3.00 (2.98,3.02)	2.93 (2.89,2.96)	3.07 (3.04,3.09)

^*^Unadjusted. †Number of in-hospital days and emergency department visits. ‡Covariate values: sex, female; age in decades, 5.83; ASA grade, I/II; and Charlson co-morbidity index, 0.

In the analysis of relative variable importance, smoking status had a Shapley value of 0.007, Wald X of 9.918, and Wald X (%) of 0. Time spent in the operating room and urgency class were the most important variables (*Table S3*).

## Discussion

In this retrospective cohort study on 90-day postoperative healthcare costs associated with smoking, current smokers have significantly increased costs compared with never smokers.

Kamath *et al*.^9^ were the first to explore the association between current and former smoking and perioperative and postoperative healthcare costs; they measured 30-day costs in a veteran population undergoing general surgery. Significantly increased costs were found among current smokers compared with never smokers. Kamath *et al*.^9^ imputed missing data to avoid elimination of patients from the analyses, thereby aiming to reduce the risk of selection bias. In the present study, costs are measured at 90 days after surgery and all surgical fields are considered. The longer follow-up interval may have allowed more comprehensive cost detection.

Warner *et al*.^10^ measured perioperative and postoperative healthcare costs in a population of American patients who underwent surgery. The adjusted costs during the postoperative year were significantly higher among both current and former smokers compared with never smokers, although there was no significant difference in adjusted costs at index hospitalization. The strengths of the study of Warner *et al*.^10^ were the broad range of surgical procedures, the diverse patient sample, and the long follow-up time that allowed capture of both early and late healthcare costs.

In the present study, time spent in the operating room and urgency class are the most important cost contributors. It is a logical finding that increased surgical complexity and patient morbidity correlate with increased costs. Although the role of smoking is modest in the relative variable importance model, it is important to note that smoking status is the only modifiable variable.

The main strengths of the present study are the large, real-world sample and inclusion of all surgical specialties. The cost units utilized for total cost determination comprehensively consider charges associated with hospitalization and inpatient care. The 90-day follow-up interval enables the capture of both early and late postoperative costs.

The study also has some limitations. Due to the lack of more comprehensive cost data, the cost estimations only account for inpatient days spent on a ward and emergency department visits and not for costs of the surgeries themselves, ICU admissions, or indirect costs in the late postoperative interval (such as medications, sick leave, healthcare centre visits, and rehabilitation). This makes the cost estimations rather conservative; the cost estimates are based on mean costs across only five Finnish university hospitals, weakening the applicability of the results. The analyses cannot be stratified by pack-years smoked as they are not routinely registered in the electronic health record. As no imputation methods were used, patients meeting the exclusion criterion of having any missing data could not be included in the final sample. This may lead to some degree of selection bias. Indeed, the inconsistent recording of preoperative smoking status poses problems for both medical professionals and researchers.

Based on the results of the present study, smoking significantly increases postoperative healthcare costs. Although the excess postoperative healthcare costs of a single smoker are small, the total costs caused by smoking are considerable at the hospital district and national levels. Future studies aiming to develop effective and feasible preoperative smoking cessation interventions and further exploring their potential cost-effectiveness are of great importance.

## Supplementary Material

zrae090_Supplementary_Data

## Data Availability

The data sets generated and analysed during the study are not publicly available, but are available from the corresponding author upon request.
