# Supplementary material for: Attribution of smoking to healthcare costs in the postoperative interval
Source: BJS Open. 2024 Aug 16;8(4):zrae090. doi: 10.1093/bjsopen/zrae090 (PMC11327870; doi:10.1093/bjsopen/zrae090)
Supplement: zrae090_Supplementary_Data [file zrae090_supplementary_data.docx]

**The attribution of smoking to healthcare costs in the postoperative period**

Authors: Helene L Gräsbeck, MD, PhD student^1,2^, Aleksi R P Reito, MD, PhD, Assoc. Prof.^3,4^, Heikki J Ekroos, MD, PhD, Chief Physician^1^, Juhani A Aakko, D.Sc. (Tech.), Data Scientist^5^, Olivia Hölsä, M.Sc. (Tech.), Data Scientist^5^, Tuula M Vasankari, MD, PhD, Professor^6,7^

^1^ Pulmonary Unit, HUS Porvoo Hospital, Porvoo, Finland

^2^ Doctoral Programme of Clinical Research, University of Helsinki, Helsinki, Finland

^3^ Center for Musculoskeletal Diseases, Tampere University Hospital, Tampere, Finland

^4^ Faculty of Medicine and Health Technology, Tampere University, Tampere, Finland

^5^ Medaffcon Oy, Espoo, Finland

^6^ Department of Pulmonary Diseases and Clinical Allergology, University of Turku, Turku, Finland

^7^ Finnish Lung Health Association (Filha), Helsinki, Finland

**Corresponding author:** Helene L Gräsbeck, Pulmonary Unit, HUS Porvoo Hospital, Sairaalantie 1, 06150 Porvoo, Finland. helene.grasbeck@hus.fi **ORCID ID** 0000-0002-6297-8165

**Supplementary Materials - Index**

| **Supplementary Methods** |  |
| --- | --- |
| Exclusion criteria | *page 2* |
| Healthcare cost data  Definition of smoking status  Statistical analysis | *page 2*  *page 2*  *page 2–3* |
| **Supplementary Results** |  |
| Detail | *page X* |
| Detail | *page Y* |
| **Supplementary Appendices** |  |
| Detail | *page X* |
| Detail | *page Y* |
| **Supplementary Figures and Tables** |  |
| Supplementary Figure 1 | *page 6* |
| Supplementary Table 1  Supplementary Table 2  Supplementary Table 3 | *page 7*  *page 8*  *page 9* |
| **References** | *page 10* |
|  |  |

**Supplementary Methods**

*Exclusion criteria*

Exclusion criteria were age below 16 years, non-surgical procedure, unknown smoking status, American Society of Anesthesiologists (ASA) class 6, and missing data. 90-day reoperations were excluded due to expected overlaps between the costs of the index and subsequent procedures.

*Healthcare cost data*

The outcome was calculated based on the number and cost of 90-day postoperative inpatient days and emergency department visits. The calculations were based on unit costs reported by Mäklin et al.^1^ (see ‘Liitetaulukko 3, yliopistolliset sairaalat’). For this unit-cost report, data had been gathered from all five Finnish University Hospitals regarding numbers of hospitalizations, their mean costs, and the mean number of inpatient days. The hospital stays included medical examinations, procedures, medications, and hospitalization charges. Costs included workforce, material and supply costs, and direct and general costs of machines and devices. Since the sample included day surgery procedures, the mean cost of one in-hospital day was added to each operation so that no patient costs would equal zero.

*Definition of smoking status*

The patients’ preoperative smoking statuses were classified with the aid of a machine learning algorithm (MLA) developed by authors O.H., J.A. and H.G. for a previous study^2^. A current smoker was defined as having smoked during the past six months, a former smoker as having stopped smoking at least six months previously, and a never-smoker as never having smoked. An unknown smoking status class was included for those whose smoking status could not be determined. Further details on the MLA development can be found in O.H.’s master’s thesis^3^.

*Statistical analysis*

The impact of preoperative smoking status (never-smoker, former smoker, and current smoker) on costs, in-hospital days, and emergency department visits was assessed. The costs were assessed using three gamma regression models with a log-link function.

The first model (model 1) was unadjusted, with costs as a dependent variable and smoking status as an independent variable. The second model (model 2) included the costs and smoking status as the dependent and independent variables, respectively, but was additionally adjusted with age as a continuous covariate and sex, Charlson Comorbidity Index (CCI) and ASA class as categorical covariates. In model 3, in addition to the model 2 covariates, time spent in operating room was included as a continuous covariate, and urgency class and anaesthesia type as categorical covariates. Surgeries with missing covariate values were omitted.

Corresponding unadjusted and adjusted regression coefficients with 95% confidence intervals were calculated. The relative importance of the variables in the model was assessed by two complementary methods. The first method is based on the workflow by Harrell^4^, where the variable importance is defined by calculating the partial chi-square statistic (Wald X) for each variable. As a second method, we used a global importance score based on Shapley values (absolute log odds scale) which is a game-theoric approach to assign an importance value to each variable^5^. Shapley values were calculated as implemented in the fastshap R package^6^. Higher values (Wald X or Shapley) indicate a greater variable importance in the model, and variables which fail to add value to the model have parameters equalling zero.

To provide a real-world interpretation of the data, predicted costs and number of events (in-hospital days and emergency department visits) were simulated for never-smokers, former smokers and current smokers by inputting the median values for the continuous variables and mode for the categorical variables in the models. Predictions were also simulated for different combinations of smoking status and urgency classes. Confidence intervals for the predictions were calculated by bootstrapping (10 000 samples).

Data processing and statistical analysis were performed using R (version 4.2.3)^7^.

**Supplementary Results**

**Supplementary Appendices**

**Supplementary Figures and Tables**

*Supplementary Figure 1. Sample selection.* Study flow diagram displaying the total number of procedures in the retrieved data, the number of procedures excluded at each step of the selection process, and the number of surgeries included in the final sample.


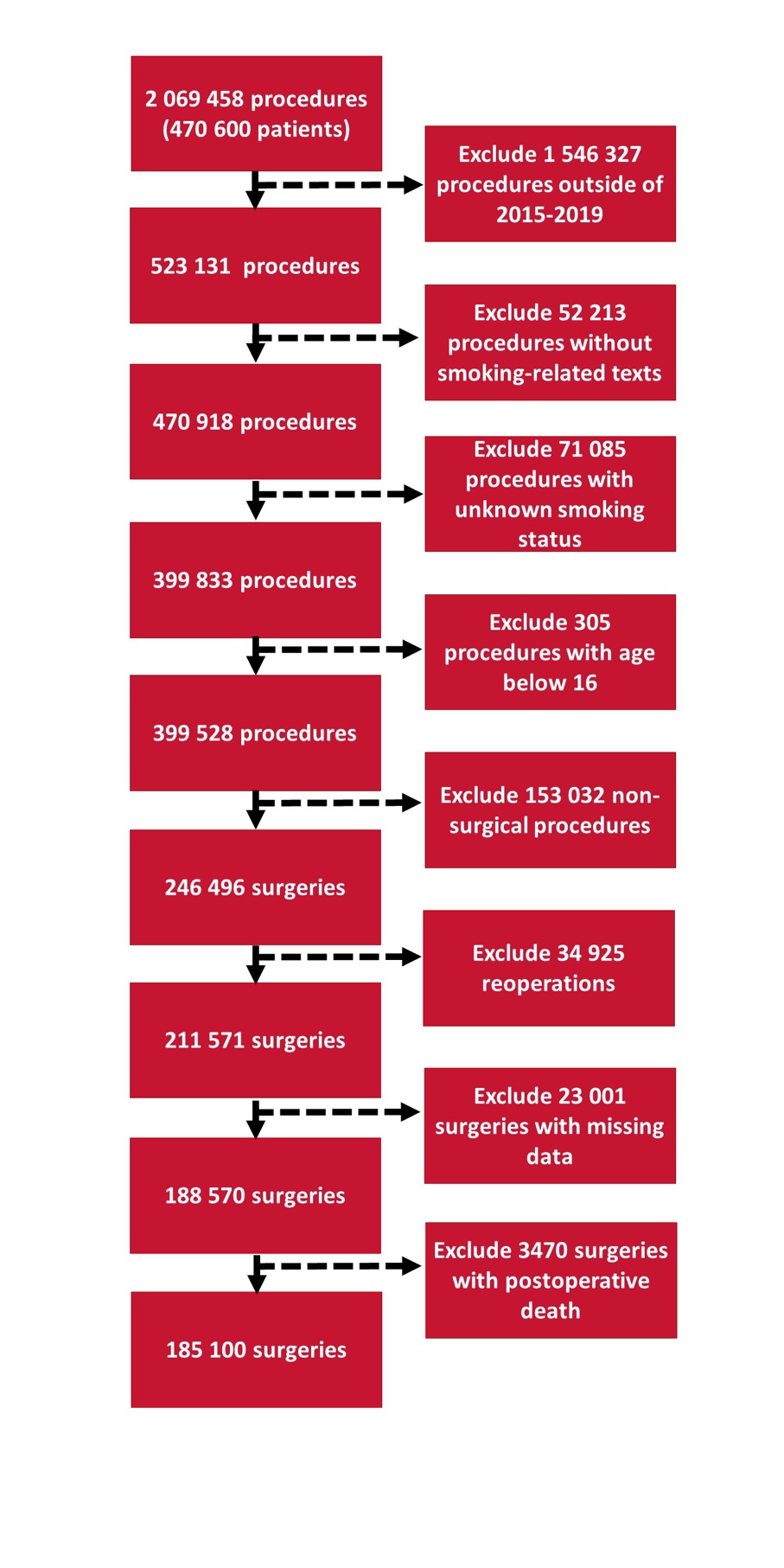


| **Supplementary Table 1.** Odds of increased postoperative healthcare costs by smoking status | | | |
| --- | --- | --- | --- |
|  | **Smoking status, OR (95% CI)** | | |
|  | Never-smoker | Former smoker | Current smoker |
| Model 1^*^ | 1 (Reference) | 1.24 (1.22-1.26), p<0.001 | 1.10 (1.09-1.11), p<0.001 |
| Model 2^†^ | 1 (Reference) | 0.98 (0.96-0.99), p<0.001 | 1.03 (1.02-1.04), p<0.001 |
| Model 3^‡^ | 1 (Reference) | 1.01 (1.00-1.02), p=0.166 | 1.02 (1.01-1.03), p=0.002 |
| ^*^ Unadjusted  ^†^Adjusted for age, sex, American Society of Anesthesiologists class and Charlson Comorbidity Index  ^‡^ Adjusted for model 2 covariates + urgency class, anaesthesia type, time spent in operating room | | | |

| **Supplementary Table 2.** Predicted total costs by urgency class and smoking status | | | | |
| --- | --- | --- | --- | --- |
|  |  | Urgency class | | |
| Smoking status |  | Elective | Urgent | Emergency <24 h |
| Never-smoker^*^ | Costs (€) (95% CI) | 2458 (2435-2481) | 3371 (3329-3411) | 5398 (5330-5469) |
|  | Difference (€) (95% CI, p-value) | - | 912 (877-948, p<0.001) | 2941 (2879-3003, p<0.001) |
|  | Number of events^†^ (95% CI) | 2.40 (2.38-2.42) | 3.23 (3.20-3.26) | 5.12 (5.07-5.17) |
| Former smoker^*^ | Costs (€) (95% CI) | 2478 (2447-2511) | 3399 (3348-3451) | 5443 (5360-5534) |
|  | Difference (€) (95% CI, p-value) | 21 (-6-49, p=0.130) | 941 (892-991, p<0.001) | 2986 (2904-3073, p<0.001) |
|  | Number of events^†^ (95% CI) | 2.41 (2.38-2.44) | 3.25 (3.22-3.29) | 5.15 (5.08-5.21) |
| Current smoker^*^ | Costs (€) (95% CI) | 2499 (2468-2532) | 3428 (3379-3477) | 5489 (5409-5572) |
|  | Difference (€) (95% CI, p-value) | 41 (14-69, p=0.003) | 969 (922-1019, p<0.001) | 3032 (2952-3112, p<0.001) |
|  | Number of events^†^ (95% CI) | 2.41 (2.39-2.44) | 3.26 (3.22-3.29) | 5.15 (5.09-5.21) |
| ^*^ Covariate values: Sex: female; age in decades: 5.83; American Society of Anesthesiologists class: 1\|2; Charlson Comorbidity Index: 0; time spent in operating room in hours: 1.97; anesthesia type: general anaesthesia  ^†^ Number of in-hospital days and emergency department visits | | | | |

| **Supplementary Table 3.** Relative variable importance in prediction of total costs | | | | | | | | | |
| --- | --- | --- | --- | --- | --- | --- | --- | --- | --- |
|  | Variable | | | | | | | | |
| Statistic | Smoking status | Sex | Age | ASA | CCI | Urgency class | Anaesthesia type | Operative time |  |
| Shapley | 0.007 | 0.023 | 0.115 | 0.209 | 0.049 | 0.282 | 0.068 | 0.384 |  |
| Wald X | 9.918 | 102.349 | 1339.296 | 5874.318 | 350.942 | 14 998.969 | 3023.100 | 26 113.423 |  |
| Wald X (%) | 0 | 0.2 | 2.6 | 11.3 | 0.7 | 28.9 | 5.8 | 50.4 |  |
| Abbreviations: ASA, American Society of Anesthesiologists; CCI, Charlson Comorbidity Index | | | | | | | | | |

**References**

1. Mäklin S, Kokko P. *Terveyden- Ja Sosiaalihuollon Yksikkökustannukset Suomessa Vuonna 2017*.; 2021. Accessed September 29, 2023. https://urn.fi/URN:ISBN:978-952-343-493-6

2. Gräsbeck HL, Reito ARP, Ekroos HJ, Aakko JA, Hölsä O, Vasankari TM. Smoking is a predictor of complications in all types of surgery: a machine learning-based big data study. *BJS Open*. 2023;7(2).

3. Hölsä O. *Machine Learning-Based Classification of Clinical Notes to Extract Smoking Status from Electronic Health Records*. Thesis, School of Science, Aalto University, 2022.

4. Harrell F E Jr. *Regression Modeling Strategies With Applications to Linear Models, Logistic and Ordinal Regression, and Survival Analysis* . Second Edition. Page 186. Springer International Publishing Switzerland; 2015.

5. Lundberg SM, Allen PG, Lee SI. A Unified Approach to Interpreting Model Predictions. Accessed June 6, 2024. https://github.com/slundberg/shap

6. Greenwell B. fastshap: Fast Approximate Shapley Values. Published December 6, 2021. Accessed February 24, 2022. https://github.com/bgreenwell/fastshap

7. R Core Team. *R: A Language and Environment for Statistical Computing*. R Foundation for Statistical Computing; 2020. Accessed February 24, 2022. https://www.R-project.org/
